# Influence of Pore Size on the Acoustic Absorption Properties of Open-Cell AlSi Porous Cylinders

**DOI:** 10.3390/ma19050989

**Published:** 2026-03-04

**Authors:** Constantin Cristian Andrei, Constantin Stelian Stan, Marius Deaconu, Catalin Pirvu, Alina Dragomirescu, Iuliana Corneschi, Iuliana Stan

**Affiliations:** 1 Faculty of Materials Science and Engineering, National University of Science & Technology POLITEHNICA Bucharest, 060042 Bucharest, Romania; iuliana.corneschi@stud.sim.upb.ro (I.C.); iuliana.stan@upb.ro (I.S.); 2 Acoustics and Vibrations Laboratory, National Research and Development Institute for Gas Turbines—COMOTI, 061126 Bucharest, Romania; marius.deaconu@comoti.ro; 3 National Institute for Aerospace Research “Elie Carafoli”, 061126 Bucharest, Romania; pirvu.catalin@incas.ro (C.P.); dragomirescu.alina@incas.ro (A.D.)

**Keywords:** aircraft noise mitigation, open-cell AlSi alloy, pore diameter, porosity, impedance tube, sound absorption coefficient, acoustic impedance

## Abstract

Airframe noise generated at wing trailing edges and high-lift devices, such as flaps, remains a major challenge during landing, with significant contributions in the low-frequency band of 500–1500 Hz. While solid surfaces reflect this acoustic energy, metallic porous materials can effectively absorb it through viscous and thermal dissipation within their internal pore structure. To address this, the present study examines the acoustic absorption characteristics of open-cell AlSi porous cylinders featuring controlled pore diameters between 0.3 mm and 2.25 mm. Measurements were conducted in an acoustic impedance tube according to the ISO 10534-2:2023 standard, using six cylindrical samples (28 mm diameter, 70 mm length). Two sets of measurements were performed for each sample (front and rear faces), and the average values were used. The findings indicate that the normal-incidence sound absorption coefficient α rises as pore size increases, reaching 0.93–0.97 at low frequencies of 500–700 Hz for the samples with the largest pores (1.8–2.25 mm). These results indicate that open-cell AlSi alloys offer strong low-frequencies sound absorption, positioning them as promising options for aeroacoustic noise mitigation, including applications such as porous trailing edge and hybrid flap designs.

## 1. Introduction

The regulation of aircraft noise started in the late 1960s and early 1970s, driven by growing public concern regarding aviation’s environmental effects. The International Civil Aviation Organization (ICAO), operating as a UN specialized agency, took a leading role in creating worldwide standards for aircraft noise certification [1]. In 1971, ICAO introduced Annex 16 to the Convention on International Civil Aviation, named “Environmental Protection—Aircraft Noise”, establishing the first global noise rules for new aircraft types [1]. This step represented a major move toward coordinated international management of aviation noise.

From its start, Annex 16 has seen many revisions and updates reflecting continuous advancements in noise control technology and changing environmental priorities. The initial rules, commonly called “Chapter” standards (e.g., Chapter 2 or Chapter 3), mainly targeted engine noise and required steadily quieter aircraft. The United States’ Federal Aviation Administration (FAA) followed a similar path with its “Stage” system, where Stage 3 aircraft were noticeably quieter than earlier generations [2]. The ICAO Committee on Aviation Environmental Protection (CAEP) develops and proposes these standards, carrying out thorough technical reviews to confirm their practicability and effectiveness [1]. CAEP sets noise technology targets and assesses the environmental gains of suggested measures, frequently forecasting noise reductions over several decades.

European efforts, including the European Commission’s Flightpath 2050 plan, support ICAO’s worldwide work by defining challenging regional goals [3]. This plan provides a long-term strategy for European aviation, aiming to cut perceived aircraft noise by 65% compared to year 2000 levels [3]. Initiatives like Clean Sky, supported by the European Union, have played a key role in promoting research and new ideas for quieter aircraft, covering both engine and airframe noise reduction [4]. Together, these regulatory and research efforts encourage ongoing progress in aircraft acoustic design, pushing science and engineering to support sustainable aviation.

Over time, major improvements have reduced engine noise through better turbofan designs, higher bypass ratios, advanced acoustic liners and improved fan blades [5]. These technological innovations substantially decreased the noise generated by aircraft engines, particularly during take-off phase. However, as engine noise levels have decreased, airframe noise has become the main source of overall aircraft noise, especially during landing phase [5,6]. This phenomenon, often referred as “airframe noise issue”, shows how the main sources of aircraft noise have shifted [7].

Airframe noise originates from the non-engine components of the aircraft, mainly the high-lift devices (flaps and slats), landing gear and wing training edges [6,8]. The components interact with the airflow, creating turbulent structures and associated sound emissions. The mechanisms of airframe noise generation are complex, involving phenomena such as flow separation, vortex shedding and boundary layer turbulence [8]. For instance, extending the flaps and slats during landing significantly alters the wing’s geometry, leading to increased aerodynamic drag and the generation of broadband noise due to the interaction of turbulent flow with sharp edges and cavities [9].

These aerodynamic interactions generate broadband noise that is difficult to mitigate using conventional methods [6]. Unlike engine noise, which can be reduced through specific design changes and acoustic liners, airframe noise is closely tied to the aircraft’s shape and function [5]. Effective solutions must therefore use new approaches that reduce noise generation without compromising the aerodynamic performance, structural strength or safety [6]. This need led researchers to explore advanced material and concepts, such as the porous materials, to address this persistent challenge [10].

One of the most effective ways to reduce airframe noise, especially from high-lift devices and wing trailing edges, involves applying porous materials to aerodynamic surfaces [10,11]. These materials are designed to dissipate acoustic energy by converting sound waves into heat through viscous and thermal losses inside their complex pore structure [12]. When sound waves pass through the porous material, air particles oscillate and interact with the solid structure, creating frictional losses (viscous effects) and heat exchange (thermal effects) at the pore walls [10]. This energy conversion is the fundamental mechanism of sound absorption in porous media [12].

By integrating porous sections into components like trailing edges or hybrid flaps, pressure fluctuations and turbulent boundary layer noise can be reduced, leading to significant noise reduction while maintaining aerodynamic efficiency [11]. The porous structure acts as a “flow conditioner”, altering the boundary layer turbulence and reducing vortex shedding, which are primary sources of aerodynamic noise [13]. The acoustic performance of porous materials depends heavily on their microstructure, including parameters, such as porosity (the void volume fraction), pore size distribution, tortuosity (the flow path complexity) and flow resistivity (airflow resistance) [10,14]. Adjusting these features is essential for matching the material’s absorption to specific frequency ranges and applications [14].

Moreover, the metallic porous materials, especially those based on aluminum alloys, combine excellent mechanical strength, stiffness, thermal stability and fire resistance, making them ideal for aerospace structure use [15,16]. Their good strength-to-weight ratio also helps reduce aircraft weight and improve fuel efficiency. Although acoustic properties of polymers and ceramics have been widely studied [17], there is still limited research on open-cell AlSi alloys produced industrially. This material class, due to its production flexibility and ability to control microstructure, holds strong potential for advanced aeroacoustic applications [12].

The present study aims to provide an experimental investigation of acoustic absorption behavior in open-cell AlSi porous cylinders with precisely controlled pore diameters. The mail objective is to determine how variations in pore diameter affect the sound absorption coefficient across the low- to mid-frequency range (500–6500 Hz). By using industrially manufactured AlSi alloys, this research offers practical insights into their potential for the next-generation aircraft noise reduction technologies, such as advanced porous trailing edges and innovative hybrid flap designs. The results are expected to contribute to the development of more effective and long-lasting acoustic solutions for sustainable aviation.

## 2. Materials and Methods

This experimental investigation involved six cylindrical samples fabricated from open-cell AlSi alloy, prepared specifically for acoustic absorption testing. AlSi alloys were selected because of their favorable mechanical properties, such as high stiffness-to-weight ratio, good thermal stability and corrosion resistance, all important for aerospace applications [18,19]. Furthermore, the open-cell structure is essential for effective sound energy dissipation, allowing sound waves to enter and interact with the internal porous network [14].

### 2.1. Material Description

The cylindrical specimens were provided by Alupor LLC (Tbilisi, Georgia), a manufacturer specialized in advanced porous metallic materials [20]. Each cylinder possessed a diameter of 28 mm and a length of 70 mm. This length was selected based on the minimum material depth required for sound propagation to fully develop viscous and thermal dissipation mechanisms inside the open-cell porous media. According to porous acoustics theory, the effective penetration depth of airborne sound into rigid-frame porous materials increases at low frequencies and is governed by flow resistivity, tortuosity and pore distribution [21]. Therefore, materials with relatively small pore diameters (0.3–1 mm), such as those investigated here, require a larger interaction path length to exhibit their characteristic dissipative behavior [12]. A smaller sample thickness may underestimate the low-frequency absorption due to insufficient propagation distance within pore network, while ISO 10534-2:2023 [22] limits the maximum sample size based on tube geometry.

Considering these considerations, a length of 70 mm was selected as an appropriate compromise to capture the full dissipative response of the material across the 500–6500 Hz range, while remaining compatible with the impedance tube dimensions.

The pore diameter for each cylinder is listed in Table 1.

This range was deliberately selected to explore the transition from viscous-dominated to permeability-dominated acoustic behavior, providing insights into the optimal microstructural parameters for broadband sound absorption.

#### Manufacturing Process

The manufacturing process employed by Alupor LLC involves advanced casting techniques, such as investment casting or replication casting [20]. This process creates a highly permeable, durable material that serves as an efficient alternative to sintered metals, traditional aluminum foams and metal sponges. It allows for complex shapes, including hybrid parts combining porous and solid sections and enables customization based on pore size by varying the space-holder granules.

**Figure 1:**
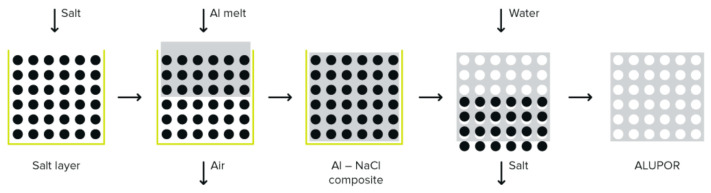
ALUPOR porous structure obtained by replication technique [20].

The replication casting method, highlighted in Figure 1, involves the following key stages, provided by Alupor LLC [20]:

1. Preparation of the space-holder mold:
  - A granular space-holder material, typically salt crystals, is selected and packed into a preheated mold.
  - The granule size determines the final pore size. This creates a temporary “scaffold” that defines the porous structure.
2. Molten metal infiltration:
  - Molten aluminum alloy (heated to approximately 710 °C) is poured into the mold under vacuum conditions to ensure complete infiltration around and between the salt granules. Vacuum casting minimizes defects, allowing the liquid metal to replicate the granular voids precisely.
3. Solidification:
  - The filled mold is allowed to cool and solidify, forming a solid aluminum structure embedded with salt granules. This step preserves the open-cell architecture, ensuring the interconnectivity of pores.
4. Removal of space-holder:
  - The solidified block is immersed into water or a leaching solution to dissolve and remove the salt granules, leaving behind an interconnected network of open pores.
  - This chemical removal process avoids mechanical damage to the delicate porous structure.
5. Post-processing and Finishing:
  - The resulting porous block is dried, inspected and machined if needed.

### 2.2. Macroscopic Analysis

The macroscopic analysis of the cylinders, performed at National Institute for Aerospace Research “Elie Carafoli”—INCAS confirmed the pore connectivity, showing that the pores form irregular and continuous channels across the entire specimen. The equipment involved in this analysis is Qness 60 A+ EVO Micro hardness tester (QATM, Golling, Austria), which is suitable for both hardness testing and microscopy. It has a 5 Megapixel color camera that provides reproductible and reliable results [23].

**Figure 2:**
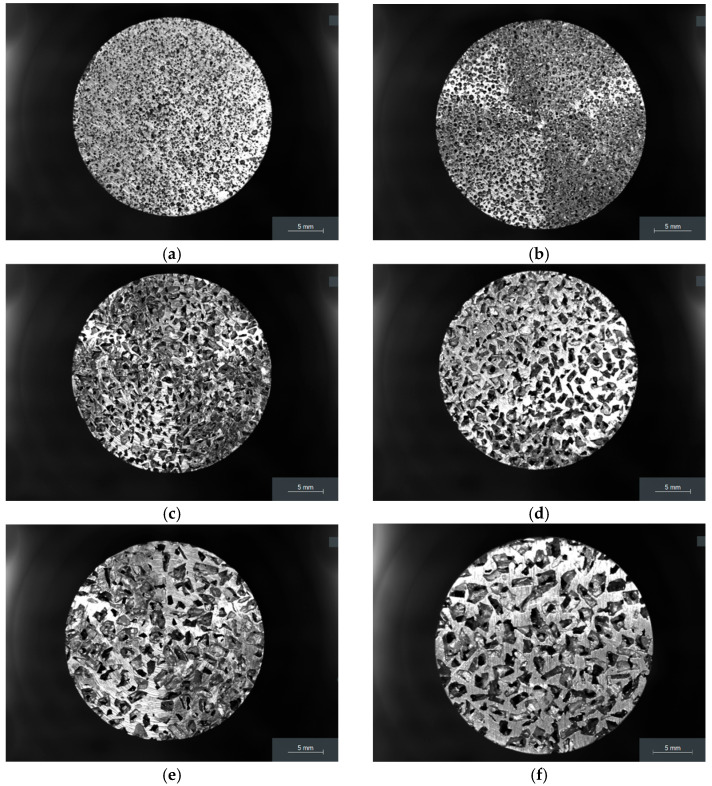
Macroscopic view of each porous cylinder: (**a**) 1st cylinder; (**b**) 2nd cylinder; (**c**) 3rd cylinder; (**d**) 4th cylinder; (**e**) 5th cylinder; (**f**) 6th cylinder.

According to Figure 2, the open-cell morphology ensures a connected network of voids that permits fluid communication throughout the material—an essential feature for acoustic absorption.

### 2.3. Chemical Composition Identification

Chemical composition of the porous AlSi alloy samples was determined using an Olympus Vanta Core handheld XRF analyzer equipped with the AlloyPlus application (Evident/Olympus, Waltham, MA, USA) [24]. The measurements were performed in MN:1.1 mode, ensuring rapid and reliable identification of major and minor elements. MN stands for metal mode (the main operating mode designed for fast and accurate analysis of metal alloys), whereas 1.1 refers to the specific version of the calibration algorithm used at the time of the measurement.

The use of this portable, next-generation XRF device allowed fast, non-destructive analysis of the porous specimens, providing consistent and reproductible results across all six samples studied. Typical composition of the porous cylinders is shown in Table 2.

The results for each element are reported as mean values together with their associated measurement uncertainty expressed as ±3σ, where σ represents the standard deviation. This uncertainty interval is calculated at a confidence level of 99.73% (corresponding to a factor of 3, according to Montgomery [25]). Thus, for each element in Table 2, the reported value ±3σ means that approximately 99.73% of all possible measurements (under the same experimental conditions) are expected to fall within this interval around the mean. In other words, there is only a 0.27% probability that a true measurement would lie outside the stated ±3σ range.

### 2.4. Porosity Analysis

The porosity was determined using two methods: (1) gravimetrically, by comparing the mass of the porous specimen with that of a solid reference cylinder of the same dimensions and (2) using CT scan.

The gravimetrical method provides an accurate measure of the void fraction within the material, a key parameter for acoustic modeling and characterization. Thus, the porosity φ for each sample was obtained from the following relation, derived from [12,15]:

(1) $$\varphi =1-\frac{m_{porous}}{m_{solid}}$$

where $m_{porous}$ and $m_{solid}$ represent the masses of the porous and solid AlSi cylinders, respectively. The mass of solid AlSi cylinder was calculated using the material density and the cylinder volume.

The density of the solid (non-porous) AlSi matrix was taken as 2640 kg/m^3^ for the calculation of open porosity in the porous cylindrical specimens. This value was selected based on the measured Si content of the alloy (~14 wt.% Si, as reported in Table 2) and on reliable literature data for Al-Si alloys. Specifically, Nikanorov et al. [26] measured the density of rapidly solidified Al-Si and reported a nearly linear decrease with increasing Si content, yielding densities of approximatively 2.62–2.64 g/cm^3^ (2620–2640 kg/m^3^) in the range of 14–15 wt.% Si.

The second method involved in this porosity analysis was CT scan using the Nikon Metrology NV equipment, model XT H 225 (Nikon Metrology, Leuven, Belgium) and Inspect-X XT software (version 6.14.4). The volumetric reconstruction was carried out using CT Agent XT (version 6.14.4). The reconstructed volumes were subsequently processed to determine porosity and the spatial distribution of pores using VGSTUDIO MAX software (version 2024.3), enabling the calculation of total porosity. The scanning parameters (Table 3) were applied to all six samples to ensure comparable results.

### 2.5. Acoustic Analysis

All acoustic measurements were conducted at the National Research and Development Institute for Gas Turbines—COMOTI, Bucharest, Romania, using a two-microphone impedance tube in accordance with the ISO 10534-2:2023 [22].

#### 2.5.1. Principle of Operation

The impedance tube method determines the sound absorption coefficient (α) and acoustic impedance (Z) of a sample by measuring the transfer function between the two microphones positioned along the axis of a cylindrical tube.

A broadband sound signal is emitted by a loudspeaker at one end, propagating through the tube until it interacts with the test specimen mounted at the opposite end.

The incident and reflected pressure waves interfere, forming a standing-wave pattern. By measuring the sound pressures $p_{1}$ and $p_{2}$ at two distinct locations, the complex reflection coefficient R and absorption coefficient α can be determined according to ISO 10534-2:2023 [22]:

(2) $$R\left(\omega \right)=\left|R\right|e^{j\phi_{R}}=\frac{H_{12}\left(\omega \right)-H_{i}}{H_{r}-H_{12}\left(\omega \right)}e^{2jk_{0}x_{1}},\;\alpha \left(\omega \right)=1-{\left|R\right|}^{2}$$

where

$x_{1}$ is the distance between the sample and the further microphone location
$x_{2}$ is the distance between the sample and the second microphone location
$\Phi_{R}$ is the phase angle of the normal incidence reflection coefficient
$H_{i}=\frac{P_{i2}}{P_{i1}}=e^{-jk_{0}\left(x_{1}-x_{2}\right)}=e^{-jk_{0}s}$ is the transfer function of the incident wave alone
$H_{r}=\frac{P_{r2}}{P_{r1}}=e^{jk_{0}\left(x_{1}-x_{2}\right)}=e^{jk_{0}s}$ is the transfer function of the reflected wave alone
$s=x_{1}-x_{2}$ is microphone spacing.

#### 2.5.2. Experimental Configuration

**Figure 3:**
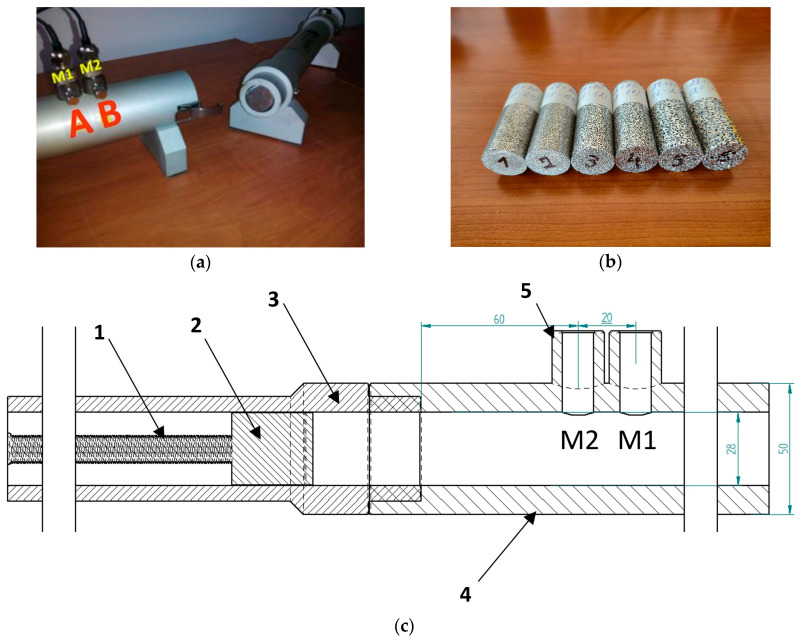
Experimental layout: (**a**) impedance tube [28]; (**b**) porous samples; (**c**) impedance tube components.

The main components involved in this experiment are:

- Microphones M1 and M2 placed in positions A and B (corresponding to x_1_ and x_2_ from Equation (2)): GRAS, ¼” type 40BP [27], serial no. 90324, serial no. 90325 (Figure 3a)
- Preamplifiers: GRAS, ¼” type G-26AC, serial no. 86048, serial no. 86091 (GRAS Sound & Vibration, Holte, Denmark)
- Acquisition system + noise generator: SYMPHONIE [26], type SSP3002000, serial no. 1884
- Power amplifier: MESA V31, type AR42AOK (MESA Engineering, Petaluma, CA, USA)
- Tube Φ 28 mm: SCS9020B/K, serial no. H1008/2-C, frequency domain 500–6300 Hz (Figure 3c)
- Speaker Φ 28 mm: SCS9020B/K, serial no. H1008/2-AP
- Calibrator: GRAS, type 42AB, serial no. 31543
- Software: Metravib dBAlphaTest, which automatically calculates the acoustic attenuation coefficient

A representation of the impedance tube setup [28] along with the AlSi porous cylinders involved in this study are provided below in Figure 3a,b. Figure 3c represents the impedance tube components, where 1 is the threaded rod for fine adjustments of the sample position, 2 is the rigid termination, 3 stands for the sample holder tube, 4 represents the microphone and loudspeaker tube and 5 is the microphones’ (M1 and M2) holder.

The measurement configuration allowed accurate determination of the absorption coefficient and the normal-incidence impedance of each sample over the full frequency range. To ensure repeatability, each test was conducted twice—once with the front face of the specimen exposed to the sound wave, and once with the sample reversed. The mean value of the two tests was used as the final result.

#### 2.5.3. Experimental Procedure

The measurements were performed using the two-microphone impedance tube system described in Section 2.5.2, compliant with ISO 10534-2 [22].

Before each measurement, the impedance tube was calibrated using an absorptive specimen to determine the reference phase and amplitude of the incident wave. The loudspeaker output was adjusted to ensure a uniform sound pressure level across the entire frequency range of interest.

Each cylindrical specimen was carefully inserted into the test section of the tube, ensuring a tight fit without leakage. The setup was allowed to stabilize for 30 s before data acquisition. The frequency sweep was recorded over 10 averages to minimize random noise.

The acoustic absorption coefficient α(f) was calculated at narrowband frequency resolutions. For each specimen, α(f) curves were plotted and compared to analyzing the effect of the pore diameter.

A similar study on open-cell Al alloy foams with graded pore sizes (0.8–2.2 mm) and porosity of 66–67% was reported by Ke et al. [12]. Their 20 mm thick specimens achieved excellent peak absorption coefficients of 0.96–0.99 in the mid-frequency range (~1600–4500 Hz), with a α dropping sharply below 1000 Hz (typically α < 0.5 at 500–800 Hz). Thus, this study aims to follow the same analysis for a 70 mm thickness: calculation of sound absorption coefficient across the low- to mid-frequency range (500–6500 Hz) for different pore sizes and evaluation of how variations in pore diameter influence the sound absorption coefficient.

#### 2.5.4. Data Processing and Error Analysis

The repeatability of the measurements was assessed by calculating the standard deviation between the two experimental runs (front and back orientations). Across all specimens and frequencies, the variation in α(f) was below ±0.05, indicating excellent repeatability.

The results were further analyzed in terms of the acoustic impedance Z, normalized by the characteristic impedance of air Z_0_. This provided insights into the resistive (real) and reactive (imaginary) components of the sound-field interaction with the porous structure.

## 3. Results

### 3.1. Porosity Results

The comparison between the porosity values obtained using the gravimetric method and those derived from CT scanning shows very small relative differences, ranging from 0.3% to 2.54%, according to Table 4. The difference was determined using the following expression:

(3) $$Difference\;\left(\%\right)=\frac{\left|Graviemtrical\;porosity-CT\;scan\;porosity\right|}{Gravimetrical\;porosity}\cdot 100$$

Such low deviations indicate a high level of agreement between the two measurement techniques. This consistency suggests that both the mass-volume approach and the three-dimensional structural characterization provided by CT accurately capture this characteristic of porous architecture of the cylinders.

The slight differences observed can be attributed to the intrinsic limitations of CT imaging, particularly its resolution, which may fail to detect very small or partially closed pores. As a result, CT measurements can occasionally underestimate the total porosity compared to the gravimetric method—a behavior well documented in the literature [29,30]. Overall, the low relative difference confirms the reliability of the results and the uniformity of the samples produced.

### 3.2. Acoustic Results

This experimental investigation examined the acoustic absorption and impedance behavior of open-cell AlSi porous cylinders with six different mean pore diameters, ranging from 0.3 mm to 2.5 mm. The specimens, produced by Alupor LLC were analyzed in the frequency range 500–6500 Hz using a two-microphone impedance tube at COMOTI Bucharest, as mentioned in Section 2.5.

#### 3.2.1. Sound Absorption Coefficient Analysis

**Figure 4:**
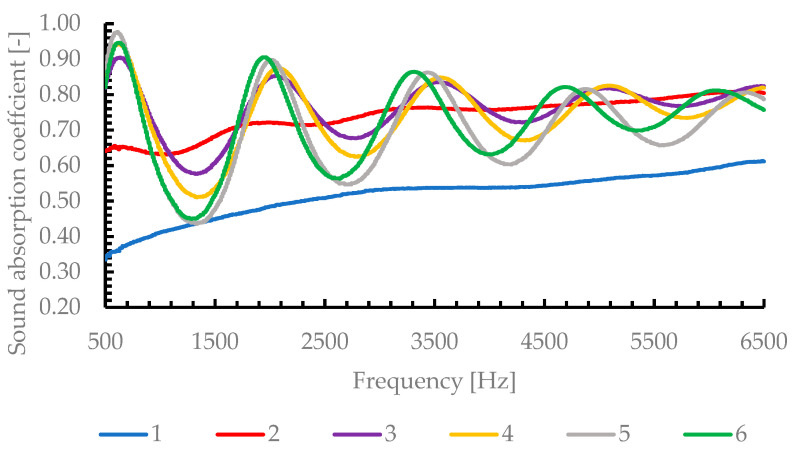
Narrow band spectrum α = α(f)—1–6 represents the samples ordered ascendingly in function of mean pore diameter (according to Figure 3b).

The experimentally obtained sound absorption coefficients α(f) for the six AlSi porous cylinders are summarized in Figure 4, which presents the averaged results (front and back faces) across the full frequency range of 500–6500 Hz. Each curve corresponds to one average pore diameter, listed in Table 1: 0.3 mm, 0.65 mm, 1 mm, 1.2 mm, 1.8 mm and 2.25 mm.

For each cylindrical specimen, acoustic measurements were performed twice—once with the front face exposed to the incident sound wave and once with the rear face exposed—to assess the influence of possible microstructural asymmetries or surface effects. The results showed excellent repeatability, with the absolute difference in the sound absorption coefficient α(f) between the front-face and rear-face measurements remaining consistently below ±0.05 across the entire frequency range of 500–6500 Hz and for all six pore diameters investigated. This small variation confirms the high uniformity of the open-cell structure throughout the cylinders and indicates that any potential anisotropy or surface irregularities have a negligible impact on the overall acoustic performance. Consequently, the final absorption coefficient curves presented in Figure 4 represent the arithmetic mean of the front- and rear-face measurements for each specimen.

The graph of the sound absorption coefficient α(f) for the six samples (numbered 1–6 in ascending order of mean pore diameter) clearly reveals two distinct behavioral regimes and several phenomena commonly observed in open-cell porous materials.

Under normal incidence conditions, the absorption coefficient is governed by the impedance matching between the material surface impedance Z_S_ and the characteristic impedance of air Z_0_.

As the pore diameter increases, the airflow resistivity decreases, allowing deeper acoustic penetration into the porous structure. This reduces the surface impedance mismatch between the material and air, leading to a lower reflection coefficient and therefore higher absorption.

In contrast, small pore sizes result in high flow resistivity, limiting acoustic penetration and increasing reflection at the surface, which explains the lower absorption values observed for samples 1 and 2.

A key observation is that the frequency of the absorption peak shifts toward lower frequencies as the pore diameter increases. This occurs because a larger pore size reduces surface impedance and effectively increases the acoustic penetration depth, leading to resonance or impedance-matching conditions at lower frequencies. Thus, larger pores couple acoustic energy more efficiently at low frequencies, while small pores are effective only at higher frequencies.

Moreover, the curves exhibit distinct dips (reductions in α) around certain bands (e.g., ~1200–1700 Hz), followed by secondary peaks (~2200–3300 Hz). These oscillations arise from the interaction between the sample’s surface impedance and standing waves within the impedance tube—a well-known effect in quarter-wave resonator configurations.

At higher frequencies, α approaches a plateau, typically between 0.7–0.8 for all samples except the smallest pores. The loss mechanisms become dominated by tortuosity and multiple scattering within the interconnected structure, typically observed in open-cell porous materials at high frequencies. The experimental data suggest that when the wavelength becomes comparable to the pore dimensions, partial reflections and local resonances occur, slightly reducing the overall absorption efficiency.

To highlight the uniqueness of the present results, Table 5 compares absorption performance (peak α, frequency range, thickness, porosity) with key studies on open-cell metallic foams from the literature [12,14,15]. The table shows that the AlSi samples with large uniform pores achieve significantly higher low-frequency absorption than previously reported materials, even at lower porosity.

The absorption coefficients of 0.93–0.97 achieved at the frequency range of 500–700 Hz are relevant for airframe noise reduction at wing trailing-edge and flaps. In these regions, vortex shedding and boundary layer turbulence, resulted from the fluid–structure interaction, produce significant pressure fluctuations in the 500–1500 Hz band. By integrating porous surfaces, the strength of these fluctuations can be reduced by allowing the airflow to pass through the porous structure, weakening the vortex formation and attenuating the resulting acoustic radiation.

#### 3.2.2. Acoustic Impedance Analysis

**Figure 5:**
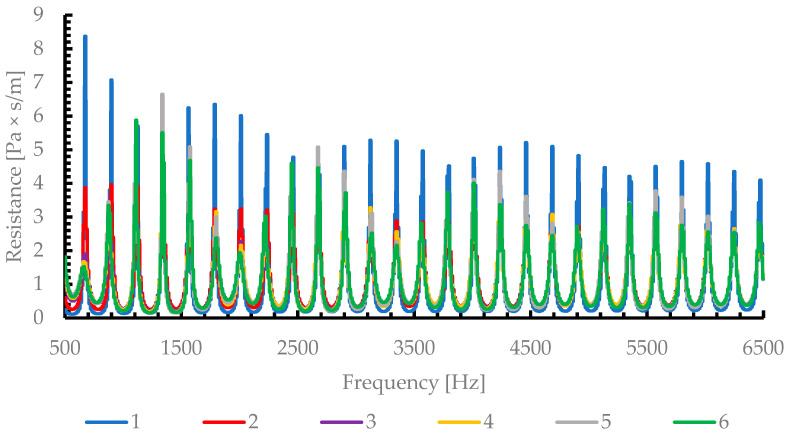
Acoustic Impedance—Resistance in function of frequency.

Figure 5 presents the variation in the acoustic resistance (real part of the impedance) as a function of frequency, for all six AlSi porous cylinders with different average pore diameters (0.3 mm, 0.65 mm, 1 mm, 1.2 mm, 1.8 mm and 2.25 mm).

A general trend can be observed: the resistance curves show that samples with smaller pore diameters (samples 1 and 2) have higher resistance peaks (up to 8–9 Pa·s/m), especially around 600–700 Hz. This implies stronger viscous dissipation and therefore higher energy loss by friction. However, excessive resistance can also lead to impedance mismatch with air, limiting sound energy transmission into the material and reducing the absorption coefficient at low frequencies—a behavior confirmed in Figure 3, where samples 1 and 2 exhibit the lowest sound absorption coefficients (α~0.3–0.6).

**Figure 6:**
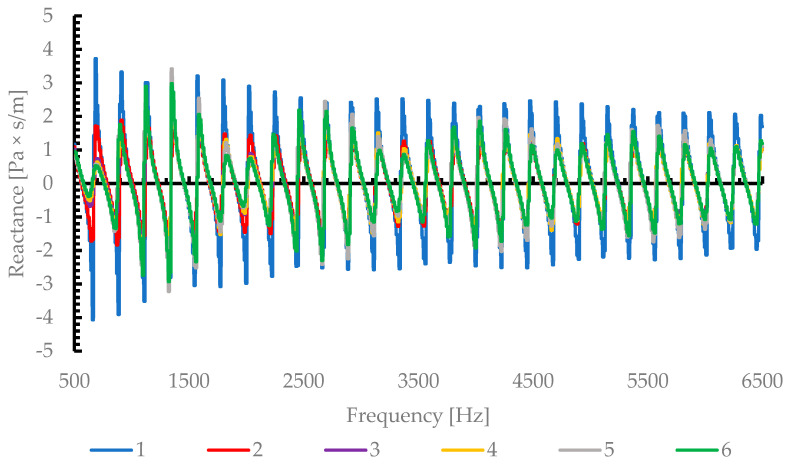
Acoustic Impedance—Reactance in function of frequency.

Figure 6 illustrates the amplitude of the reactance oscillations decreases with the increasing pore diameter, suggesting that larger pores reduce the inertial effects associated with trapped air masses in the pore channels. Consequently, the phase shift between pressure and particle velocity becomes smaller, leading to a better impedance match with the surrounding medium and thus enhanced sound absorption. This explains the smoother and more stable behavior of samples 5 and 6 (1.8 and 2.25 mm pore diameter), which corresponds to the highest absorption coefficient (α > 0.95) over a broad frequency range (500–6500 Hz).

By correlating Figure 4, Figure 5 and Figure 6, it becomes evident that the optimal acoustic performance occurs when a proper balance between resistance and reactance is achieved.

The results reveal a clear dependence of the sound absorption coefficient on pore size. The key findings can be summarized as follows:

- The absorption coefficient α increases with the mean pore diameter, reaching a maximum value of approximatively 0.97
- Materials with too high acoustic resistance (small pores) exhibit high sound energy dissipation but poor impedance matching, resulting in partial sound reflection. Thus, viscous resistance dominates, leading to limited acoustic energy penetration and relatively low absorption (α ≤ 0.54)
- A transition from viscous to inertial acoustic behavior occurs around 1 and 1.2 mm pore diameter, beyond which the absorption improves substantially and stabilizes at high values.
- The acoustic impedance analysis confirmed that optimal impedance matching (Z~Z_0_) occurs near 1.2–1.8 mm pore diameters, providing the most efficient sound energy dissipation
- Samples with too low resistance (very large pores) may allow excessive transmission, reducing energy dissipation. Thus, the absorption remains broad-band and nearly constant across the frequency range (500–6500 Hz), demonstrating the material’s potential for low-frequency noise attenuation.

Therefore, the gradual increase in the absorption coefficient with the pore diameter can be attributed to an improved acoustic impedance matching between the porous surface and air, as well as reduced inertial resistance.

This result confirms that the acoustic behavior of open-cell metallic foams, such as AlSi depends not only on porosity, but also on the characteristic pore dimension and interconnectivity, which govern the balance between viscous energy dissipation and inertial resistance.

## 4. Conclusions

The acoustic absorption behavior of open-cell AlSi porous cylinders with pore diameters ranging from 0.3 to 2.25 mm was experimentally investigated over the 500–6500 Hz frequency range, according to ISO 10534-2 [22].

Key findings include:

- Small pore diameters (0.3–0.65 mm) result in low absorption coefficients that increase gradually with frequency, due to high flow resistivity and limited sound penetration.
- Larger pores (>1.2 mm) yield significantly higher absorption, with peak values approaching unity at low frequencies of 500–700 Hz.
- The absorption coefficient α increases markedly with pore size, reaching 0.93–0.97 in the 500–700 Hz range for pores of 1.8–2.25 mm and the peaks remain above 0.7 across the full bandwidth.
- Acoustic impedance analysis shows that optimal matching (Z~Z_0_) occurs at pore diameters of ~1.2–1.8 mm, balancing viscous dissipation and inertial effects for stable broadband absorption.

This study was limited to normal-incidence acoustic measurements performed in an impedance tube, which do not replicate diffuse-field or gazing-incidence conditions typical of aeroacoustic environments. The specimens had a fixed cylindrical geometry, while practical applications, such as porous trailing edges, generally employ thin plates or tapered structures, whose geometric effects were not investigated. Additionally, parameters such as flow resistivity and permeability were not directly measured, limiting the quantitative correlation between pore morphology and acoustic impedance. Finally, no aerodynamic or aeroacoustic tests under airflow were carried out, so the interaction between the porous material and boundary layer turbulence remains to be validated experimentally.

## Figures and Tables

**Table 1:** The pore diameter for each cylinder.

No.	Pore Diameter (mm)	Average Pore Diameter (mm)
1	0.2–0.4	0.3
2	0.5–0.8	0.65
3	0.8–1.2	1
4	1.2–1.6	1.4
5	1.6–2	1.8
6	2–2.5	2.25

**Table 2:** AlSi alloy chemical composition.

Element	Average Content (wt.%)	±3σ
Al	83.92	0.34
Si	14.65	0.33
Cu	0.665	0.038
Fe	0.476	0.045
Mn	0.171	0.035
Zn	0.096	0.011
Ni	0.015	0.006
Pb	0.007	0.002
Zr	0.0018	0.0009

**Table 3:** CT scanning parameters of the XT H225 model.

Parameters	Value
Tube voltage (kV)	123
Tube current (μA)	121
Scan time (minutes: seconds)	08:40
Number of frames per projection	2
Number of images	1440
Number of projections	720

**Table 4:** The porosity for each cylinder.

Cylinder No.	Mass (g)	Gravimetrical Porosity (%)	CT Scan Porosity (%)	Difference (%)
1	64.04	43.72	44	0.64
2	49.75	56.28	56.45	0.3
3	53.05	53.38	54.37	1.85
4	51.72	54.59	55.65	1.94
5	52.43	53.92	55.12	2.22
6	52.06	54.24	55.62	2.54
No porosity	113.79	-	-	-

**Table 5:** Comparative results of acoustic absorption performance from selected literature and the present study.

Study	Pore Size (mm)	Thickness (mm)	Porosity (%)	Peak α	Peak Frequency (Hz)	Low Frequency α (500–1000 Hz)
Ke et al. [12] (graded and uniform pores for no air gap)	0.8–2.2	20	~66	0.96–0.99	~2000	Low (<0.6)
Yu et al. [15]	Not specified	20	>90	0.75–0.9	~5000	Very low (<0.4)
Carbajo et al. [14] (replicated for air cavity depth of 5 mm)	6.2	6.11	56	~1	~2700	Very low (<0.4)
This study	0.3–2.25	70	~55	0.93–0.97	~500–700	0.93–0.97

## Data Availability

The original contributions presented in this study are included in the article. Further inquiries can be directed to the corresponding author.
